# High pyrethroid/DDT resistance in major malaria vector *Anopheles coluzzii* from Niger-Delta of Nigeria is probably driven by metabolic resistance mechanisms

**DOI:** 10.1371/journal.pone.0247944

**Published:** 2021-03-11

**Authors:** Abdullahi Muhammad, Sulaiman S. Ibrahim, Muhammad M. Mukhtar, Helen Irving, Maduamaka C. Abajue, Noutcha M. A. Edith, Sabitu S. Da’u, Mark J. I. Paine, Charles S. Wondji

**Affiliations:** 1 Vector Biology Department, Liverpool School of Tropical Medicine (LSTM), Liverpool, United Kingdom; 2 Centre for Biotechnology Research, Bayero University, Kano, Nigeria; 3 Department of Biochemistry, Bayero University, Kano, Nigeria; 4 LSTM Research Unit, Centre for Research in Infectious Diseases (CRID), Yaoundé, Cameroon; 5 Department of Animal and Environmental Biology, University of Port Harcourt, Port Harcourt, Nigeria; 6 Department of Science, School of Continuing Education, Bayero University, Kano, Nigeria; Institute of Zoology Chinese Academy of Sciences, CHINA

## Abstract

Entomological surveillance of local malaria vector populations is an important component of vector control and resistance management. In this study, the resistance profile and its possible mechanisms was characterised in a field population of the major malaria vector *Anopheles coluzzii* from Port Harcourt, the capital of Rivers state, in the Niger-Delta Region of Nigeria. Larvae collected in Port-Harcourt, were reared to adulthood and used for WHO bioassays. The population exhibited high resistance to permethrin, deltamethrin and DDT with mortalities of 6.7% ± 2.4, 37.5% ± 3.2 and 6.3% ± 4.1, respectively, but were fully susceptible to bendiocarb and malathion. Synergist bioassays with piperonylbutoxide (PBO) partially recovered susceptibility, with mortalities increasing to 53% ± 4, indicating probable role of CYP450s in permethrin resistance (*χ*^2^ = 29.48, P < 0.0001). Transcriptional profiling revealed five major resistance-associated genes overexpressed in the field samples compared to the fully susceptible laboratory colony, Ngoussou. Highest fold change (FC) was observed with *GSTe2* (FC = 3.3 in permethrin exposed and 6.2 in unexposed) and *CYP6Z3* (FC = 1.4 in exposed and 4.6 in unexposed). TaqMan genotyping of 32 F_0_ females detected the 1014F and 1575Y knockdown resistance (*kdr*) mutations with frequencies of 0.84 and 0.1, respectively, while 1014S mutation was not detected. Sequencing of a fragment of the voltage-gated sodium channel, spanning exon 20 from 13 deltamethrin-resistant and 9 susceptible females revealed only 2 distinct haplotypes with a low haplotype diversity of 0.33. The findings of high pyrethroid resistance but with a significant degree of recovery after PBO synergist assay suggests the need to move to PBO-based nets. This could be complemented with carbamate- or organophosphate-based indoor residual spraying in this area.

## Background

Malaria kills approximately 400,000 people in 2018 alone, 93% of these deaths in the sub-Saharan Africa. Nigeria alone account for the highest burden of malaria, at 25% of all global cases [1]. The control of malaria vectors is reliant on the use of chemical insecticides through indoor residual spraying (IRS), long-lasting lasting insecticidal nets (LLINs) and Insecticide treated mosquito nets (ITNs) [2,3]. These interventions have reduced malaria transmission to half its level from year 2000 to 2015 [4]. However, the major threat to these recorded successes is the issue of insecticide resistance in the major malaria vectors leading to retrogression and an increase in transmission [5,6]. Adequate data gathering on the resistance profiles and mechanisms in the local malaria vector populations is a key to guide deployment of control tools using evidence-based strategies [7,8].

The major vectors of malaria in Nigeria are *Anopheles gambiae* s.s., *Anopheles coluzzii*, *Anopheles arabiensis* and the patchily distributed *Anopheles funestus s*.*s*. [9–11]. However, *Anopheles coluzzii* has become the dominant species reported in most parts of Nigeria in recent years, e.g. in the south western region [12,13], the south eastern region [14] and the northern regions [15,16]. Insecticide resistance in *Anopheles gambiae s*.*l*. has been reported in Nigeria as far back as the early 1960s. In the southern part of the country for example, resistance to dieldrin, lindane and dichlorodiphenyltrichloroethane (DDT), but susceptibility to malathion parathion, fenthion, and bromophos was reported [17]. Moreover, in Sokoto, in the northwest of the country resistance to dieldrin, DDT and benzene hexachloride (BHC) was reported early [18]. Decades after these earlier efforts, data documenting the evolution of the malaria vectors, their insecticide resistance profiles and the underlying molecular mechanisms driving the resistance are still lacking, particularly in the Niger-Delta (south-south) region of the country.

Malaria transmission is practically perennial in the coastal regions of Nigeria (including the Niger-Delta), due to the prolonged rainy season which provides *Anopheles* breeding sites year round [19–21]. This is in contrast to the northern part of the country, with shorter rainy season (3–5 months) and thus relatively lower transmission rates [19]. Unfortunately, where numerous strides have been made in determining the transmission profiles of malaria mosquitoes, insecticide resistance and the molecular mechanisms driving it in other parts of the country, e.g. in southwestern Nigeria [13,22–24] and in the north, e.g. [11,15,25], little information is available from the south-south/Niger-Delta region. Baseline information on the abundance of *Anopheles* and its distribution [26], insecticide resistance profiles [27] are available in this region, without actually going deep into studying the molecular mechanisms involved. Equally, also there is a dearth of information on the potential impact of the resistance on the malaria control tools such, as the efficacy of bed nets, as recently conducted in other parts of the country [15,28]. As well, information on the role of target-site resistance mechanisms like the 1014F/S knockdown resistance (*kdr*) mutations [29,30] is also lacking in south-south of Nigeria, compared to other parts of the country where frequencies of these mutations have been described in several studies [14–16,31].

In this work, resistance profiles of *An*. *coluzzii* from Port Harcourt, Niger-Delta, Nigeria was investigated, establishing high resistance to pyrethroids and DDT, with evidences from qPCR (overexpression of *CYP6Z2* and *CYP6M3* especially) and synergist bioassay with piperonyl butoxide implicating cytochrome P450s in driving permethrin resistance. Analysis of the voltage gated sodium channel also revealed the presence of 1014F *kdr* mutation at high frequency, whereas 1014S was not detected. The 1575Ymutation was detected but only at a very low frequency.

## Methods

### Study site and sample collection

Port Harcourt (4.8156° N, 7.0498° E), the capital of Rivers State, Nigeria lies along the Bonny Rivers in the south-south region of the country, also referred to as the Niger-Delta. It is situated in the Tropical Mangrove, with rain extending from February to December [32]. It is ever green and ever raining because only the months of January and December can be truly called dry season, as such malaria is perennial in the region due to availability of *Anopheles* breeding sites year-round [33]. The heaviest rains in Port Harcourt occur in September with an average of 367 mm while December is the driest month of the year with an average rain of 20 mm [32].

**Fig 1:**
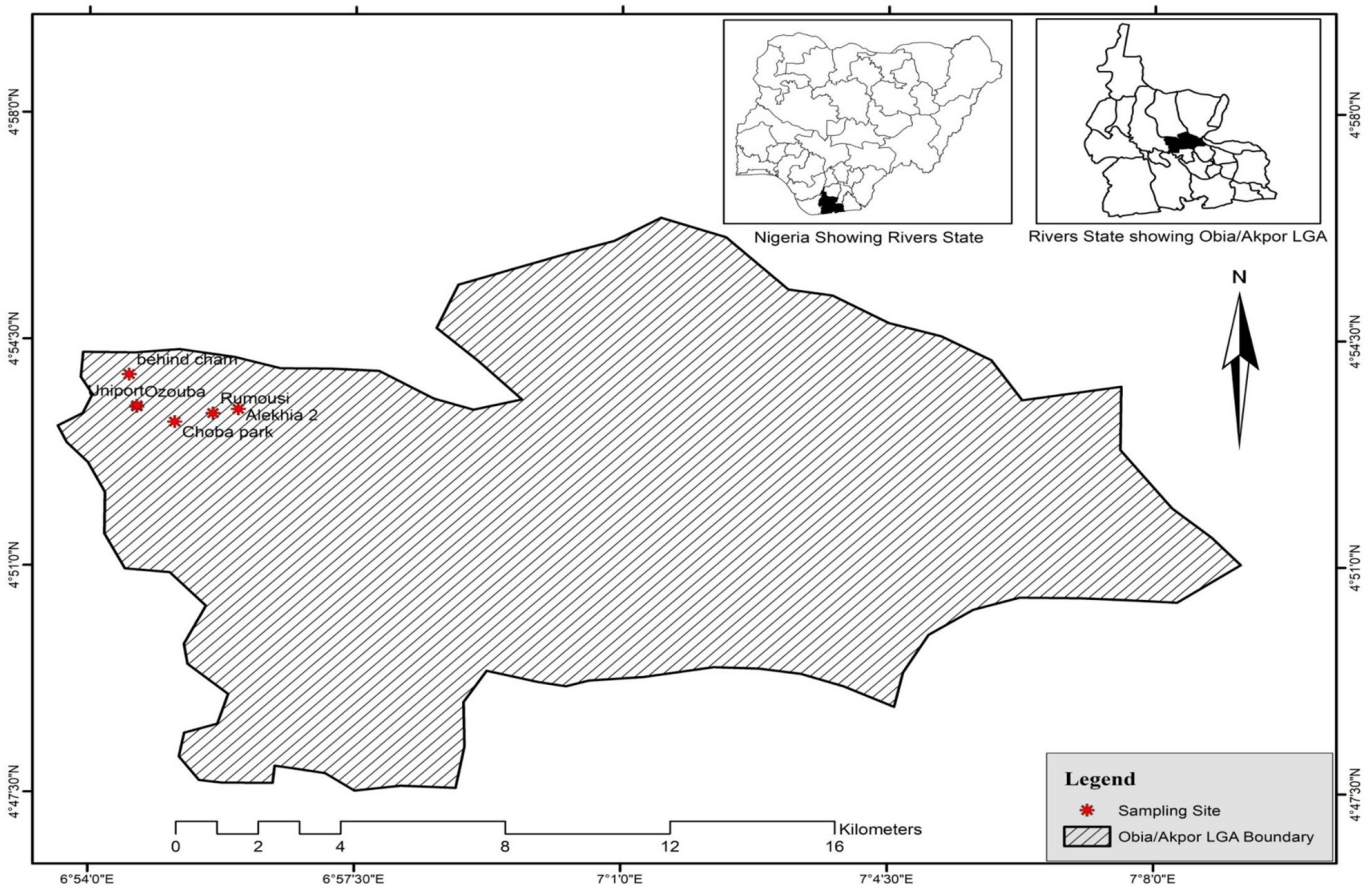
Map of sampling site, showing Obio/Akpor local Government of Port-Harcourt Metropolis, Rivers state, Nigeria.

Larval collection was conducted in Port Harcourt Metropolis in October and November 2019. Larvae were randomly collected from 33 rainwater puddles, in 5 sites, at Obio/Akpor Government Area (Fig 1). The larvae were suspected to belong to *An*. *gambiae* Complex based on their morphology (sizes and color) and all the female adults that emerged were identified morphologically as belonging to *Anopheles gambiae* s.l. [34]. Larvae were reared at the insectary (each group from particular puddle in separate tray) at the department of animal and experimental biology, University of Port Harcourt, at 25–28°C and 70–80% relative humidity.

### Mosquitoes identification to species level

Genomic DNA (gDNA) was extracted from 48 F_0_ female mosquitoes randomly selected from the cages [35]. The gDNA was utilised in the identification to the species level using the SINE200 PCR [36]. All mosquitoes were confirmed as *An*. *coluzzii* with a band of ~479 bp on agarose gel (S1 Fig). Also, legs individually pulled out from randomly selected 60 adult females used for qPCR were extracted for species identification, as above, with all the females confirmed as *An*. *coluzzii*.

### WHO insecticides susceptibility testing

The resistance profile of the mosquito population was established using the WHO protocol [37]. 3–5 days old F_0_ mosquitoes (4 replicates each of 25 females in a tube) were exposed to the diagnostic concentrations of the insecticides for 1 h. These insecticides include the pyrethroids: permethrin (0.75%) and deltamethrin (0.05%), the organochloride, dichlorodiphenyltrichloroethane (4% DDT), the carbamate, bendiocarb (0.1%), and the organophosphate, malathion (5%). All the insecticide impregnated papers were obtained from Vector Control Research Unit, University Sains Malaysia, Penang, Malaysia. One tube each was used as control for each insecticide tested, with 25 females exposed to control papers impregnated only with the carrier oil (QC controls for pyrethroids, OC control for DDT while OP and PY controls were used for bendiocarb and malathion, respectively). Knockdown rates were monitored for the pyrethroids and DDT at 15 min, 30 min, 45 min and 60 min post-exposure. Mosquitoes were then transferred into the holding tubes and allowed to recover for 24 h during which they were fed with 10% sucrose solution. Mortality was recorded 24 h after the exposure. All bioassays were carried out at at 25–28°C and 70–80% relative humidity. Resistance status of the population was established according to WHO criteria [37] for which populations with mortality > 98% are considered susceptible, population with mortality of 90–98% are considered moderately resistant, and those with mortality > 90% as resistant. Abbott’s formula was not used to correct mortalities in the control mosquitoes of which the highest was 4%. The papers were tested on a susceptible population of *An*. *coluzzii*, Ngoussou to ascertain their efficacy before being use for the testing on the field population.

### Synergist assay

Piperonyl butoxide (PBO) (obtained from Vector Control Research Unit, University Sains Malaysia, Penang, Malaysia) was utilised in a synergist bioassay with pyrethroid to investigate the potential role of P450s and/or oxidases in resistance [38]. Four replicates each of 25 F_0_ females were pre-exposed to 4% PBO for 1 h, after which they were then transferred to tubes containing 0.75% permethrin-impregnated papers, for 1 h. Mosquitoes were then transferred to holding tubes to recover for 24 h, and mortality was scored. Two tubes with 25 females were used as controls, with mosquitoes in the first tube exposed to only PBO, while those in the second tube were only exposed to permethrin [37].

### Transcriptional analysis using quantitative PCR

3–5 days old, F_0_ females which survive exposure to deltamethrin (deltamethrin-alive) and unexposed females were used for RNA extraction, alongside females from Ngousso colony. Total RNA was extracted from three biological replicates of 10 females using the Arcturus PicoPure Kit (Applied Biosystems, CA, USA) according to the manufacturer’s instructions. The RNA was treated with Dnase (Qiagen, Hilden, Germany) in order to degrade any residual gDNA. Purity and concentrations of the RNA was measured using nanodrop UV-Visible spectrophotometer (Thermo Scientific, Massachusetts, USA). 1 μg of the total RNA was used as initial template to synthesize the cDNA using an oligo(dT) primer and superscript III reverse transcriptase enzyme (Invitrogen, Waltham, CA, USA) following manufacturer’s protocol. Primers for five well-known pyrethroid metabolizing P450s, including (*CYP6P3*, *CYP6M3*, *CYP6Z2*, *CYP6Z3* [39–42] and DDT metabolizing GST, *GSTe2* [43,44] were used for qPCR (S1 Table in S1 File). Ribosomal gene S7 and glucose-6-phosphate dehydrogenase (GADH) were used as the house keeping genes [45]. 1 μl of cDNA was used as template in a PCR reaction with final volume of 20 μl. The components of the mixture included 10 μl of SyBr Green fluorescent dye (Sigma Aldrich, Germany), 0.6 μl each of the forward and reverse primers, which was topped up to 20 μl by adding 7.8 μl of nuclease free water. Thermocycling conditions were initial denaturation at 95 °C for 3 min, followed by 40 cycles each of 95 °C for 10 s, 60 °C for 10 s. Final conditions were 95 °C for 60 s, 55 °C for 30 s and 95 °C for 30 s.

The data generated were analysed according to the ddCt protocol [46] in comparison with the house keeping genes, *RSP7* (AGAP010592) and *GADH*; the efficiencies of the primers were incorporated into the calculation. The data were converted to their logarithmic forms to ensure normal distribution. The expression levels between the exposed and unexposed were analysed using tow-tailed t-test at 95% confidence levels. The list of forward and reverse primers used in the study are attached in S1 File.

### Genotyping of knockdown resistance mutations

The frequencies of the 1014F, 1014S and 1575Y voltage-gated sodium channel mutations were determined in the field F_0_ females using TaqMan assay. Genotyping was carried using 32 randomly selected females with real-time thermocyclers (Agilent, Mx3005) using a previously established protocols [47–49]. The probes for 1014F ((5’-acgacaaaatttc-3’) and 1014S (5-‘acgactgaatttc-3’) were used based on previous study [48]. Forward (5’-catttttcttggccactgtagtgat-3’) and reverse (5’cgatcttggtccatgttaatttgca-3’) *kdr* primer pairs were also adopted from the work of Bass [48]. The probes used in detecting the wild 1575Y and the N1575 were also labelled with FAM (3′nfqtttttcattgcataatagtac) and HEX (3′nfqatttttttcattgcattatagtac), and forward (tggatcgctagaaatgttcatgaca) and reverse (cgaggaattgcctttagaggtttct) primer pairs were also adopted from [49]. In a final volume of 10 μl comprise of 1 μl of gDNA, 4.25 μl ddH_2_0, 0.25 μl (40x) probes containing allelic-specific primers in each case, and 5 μl of Sensimix (Bioline, London, UK). Cycling conditions were 10 min at 95°C, followed by 40 cycles each of 92 °C for 15 s and 60 °C for 1 min. Two probes labelled with fluorescent dyes, HEX and FAM were used to detect the susceptible and resistant alleles respectively, were used. Three controls were used in each case, gDNA from known homozygote resistant, heterozygote resistant and homozygote susceptible individuals. A negative control was also used, which has 1 μl distilled water instead of gDNA. Results were analysed from scatter plots using the Mx Pro software.

### Polymorphism analysis of the Voltage-Gated Sodium Channel (VGSC)

To assess genetic diversity of the VGSC, a fragment spanning the intron-1 and intron-2, and the exon 20 was amplified in 13 deltamethrin-alive and 9 dead females This was done using the previously published set of primers *kdr*CL-F (5′-AAA TGT CTC GCC CAA ATCAG-3) and *kdr*CL-R (5′-GCA CCT GCA AAA CAA TGTCA-3) [50]. The PCR premix had 1.5 μl 10x buffer A, 0.75 μl of MgCl_2_, 0.12 μl each of Kapa Taq polymerase and dNTPs, 0.51 μl each of the forward and reverse primers, and 10.49 μl of ddH_2_0. 1 μl of the gDNA was topped up to make a total of 15 μl reaction volume. The thermal cycling conditions included an initial denaturation at 95 °C for 5 min, followed by 35 cycles each of 94 °C for 30 s, 57 °C for 30 s, 72 °C for 45 s and a final elongation at 72 °C for 10 min. PCR products were cleaned and purified using the QIAquick^®^ PCR Purification Kit (QIAGEN, Hilden, Germany) according to the manufacturer’s directives. The purified products were individually sequenced on both strands using the same pair of primers above.

For polymorphism analyses, Bioedit [51] was used to visualize and manually edit and aligned (ClustalW) the sequences. Pattern of genetic variability was assessed using the DnaSPv6.0 [52]. Maximum likelihood phylogenetic tree was constructed using MEGA 6.0 [53].

### Data analysis

Results of bioassays are presented as percentage mortalities for respective insecticides as bars with standard deviations. Two-tailed Chi-square test was conducted to compare result of synergist assays with conventional one, using GraphPad Prism (GraphPad Inc., La Jolla, CA, USA). Two-tailed t-test was also conducted to compare the expression levels between the exposed and unexposed individuals. The frequencies of *kdr* mutations were respectively calculated using the formulae F(R) = (2 x RR + RS)/2N, F(S) = 1-F(R), where RR = Total number of homozygote resistant, RS = Total number of heterozygote resistant, S = susceptible individuals and N = total number of individuals.

## Results

### Insecticide resistance profile of *An*. *coluzzii*

**Fig 2:**
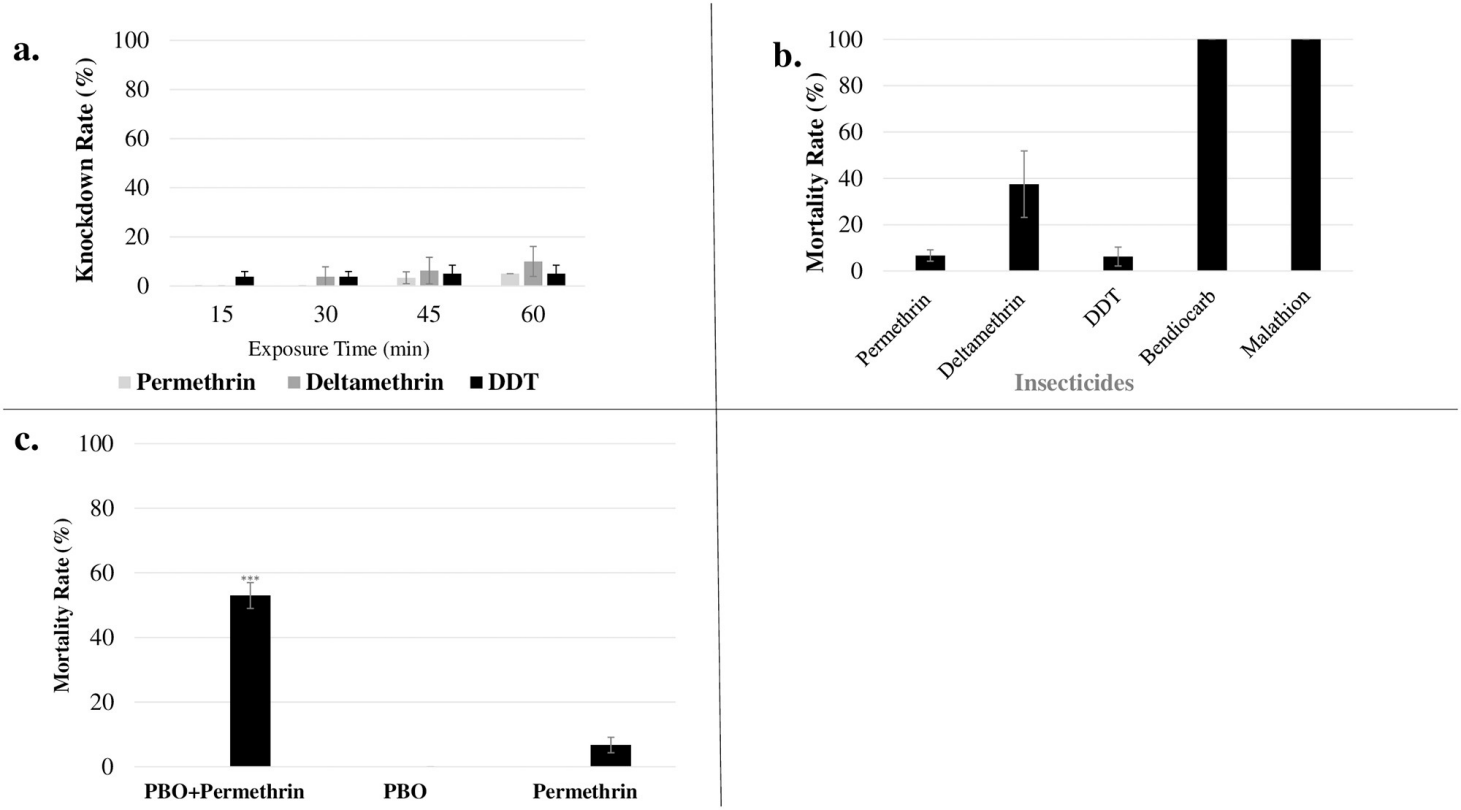
Insecticide susceptibility of the *Anopheles coluzzii* of the Niger Delta, Nigeria. **a**: Percentage knockdown rates due to exposure to various insecticides; **b**. Percentage mortalities from WHO susceptibility assay using five insecticides. Results are presented as mean ± SD (standard deviation); **c**. Result of synergist assay with PBO showing recovery of mortality in synergized mosquitoes exposed to permethrin. *** statistically significant.

Bioassay showed the Port Harcourt *An*. *coluzzii* population as highly resistant to pyrethroids and DDT. The knockdown rates in permethrin (type I pyrethroid), deltamethrin (type II pyrethroid) and DDT was very low, recorded within the range of 3.3 to the highest of 10%. These low knockdown rates were also supported by the 24 h mortalities which did not significantly increased except in the case of deltamethrin. This reiterates the fact that the population is highly resistant to the pyrethroids and DDT (Fig 2a).

A high mortality rate (37.5%) was recorded in WHO bioassays with 0.05% deltamethrin in comparison to the very low mortality of only 6.7% ± 2.4 observed with 0.75% permethrin (Fig 2b). There was no mortalities in all the negative control, except for malathion, with 4% mortality (not shown in Fig 2b). The population was more resistant to permethrin than deltamethrin. However, the organochlorine DDT also showed mortalities of only 6.3% ± 4.1, 24 h post-exposure. The population showed complete susceptibility to organophosphate malathion and the carbamate bendiocarb with a mortality of 100% for both insecticides.

### Investigating the role of P450 monooxygenases in resistance using PBO bioassay

PBO-synergist bioassay recovered some susceptibility towards permethrin with mortality increasing from 6.7% ± 2.4 as obtained from the conventional bioassay to 53% ± 4, indicating the potential role of P450s in permethrin resistance (Fig 2c). Two-tailed chi-square test indicated that the association between the recovery of susceptibility from PBO exposure as significant (*χ*^2^ = 29.479, df = 1, P < 0.0001).

### Expression profile of candidate resistance genes

**Fig 3:**
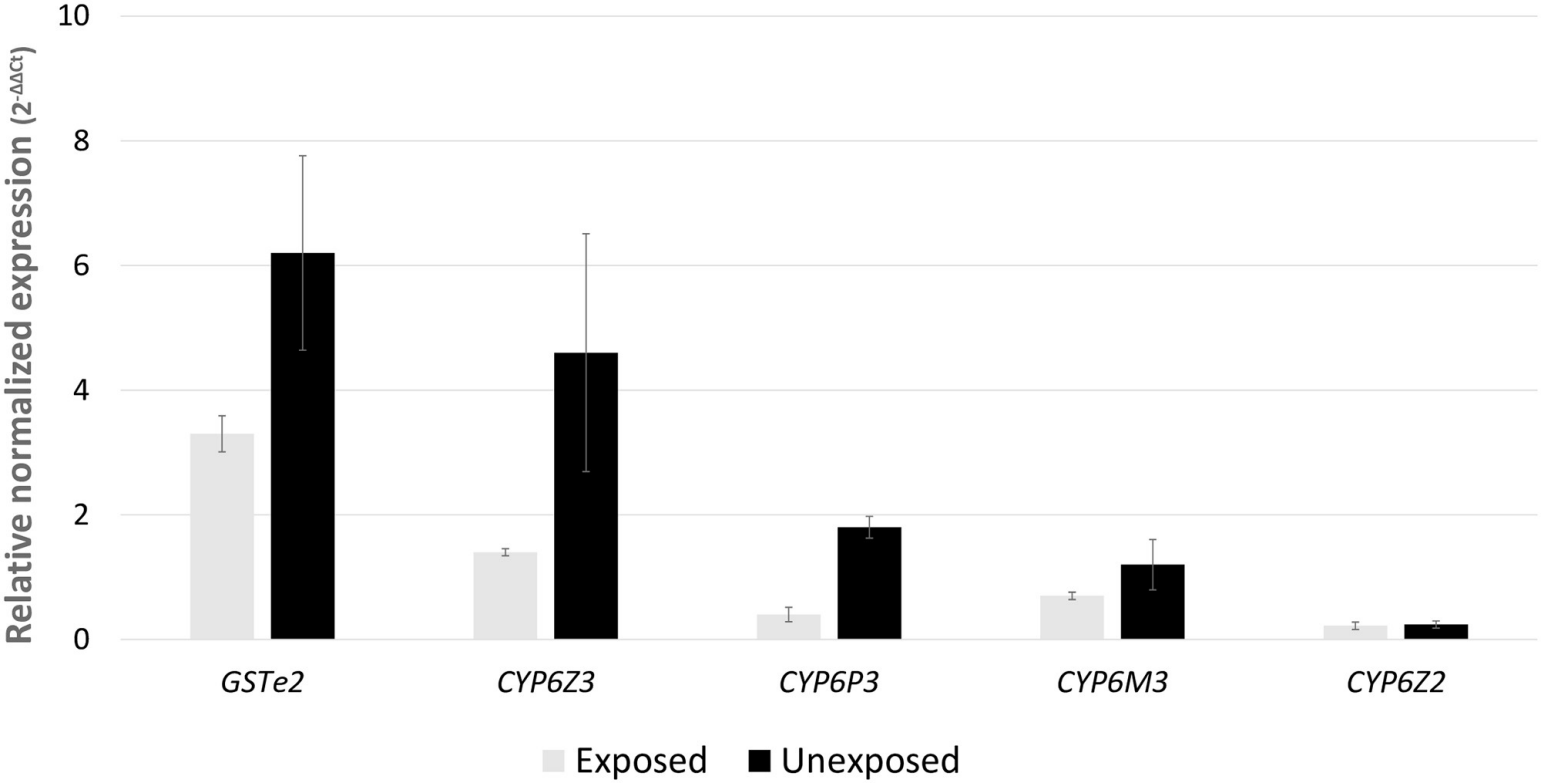
Determination of molecular basis of pyrethroid resistance in the *An*. *coluzzii* population. Results of transcription profiling of 5 major insecticide resistance gene using qRT-PCR. Values are represented as mean of expression in relation to Ngousso ± SD.

The expression of profile of four P450s and *GSTe2* was investigated in females which survived exposure, the unexposed females and Ngousso (Fig 3). These genes were found to be overexpressed in the field population compared to Ngousso. Specifically, highest expression was observed in *GSTe2* and *CYP6Z3* (Fold change = 6.2 and 4.6, respectively). The least expression was observed in *CYP6Z2* (FC = 0.24), whereas *CYP6P3* and *CYP6M3* were moderately overexpressed (1.8- and 1.2-folds). Two-tailed t-test carried out to determine the statistical differences between the expression levels in the exposed and unexposed individuals revealed expressions in the unexposed individuals to be significantly higher than the exposed for *CYP6P3* (P = 0.0025) (Fig 3).

### Investigating presence of target site resistance mutations in the VGSC

**Fig 4:**
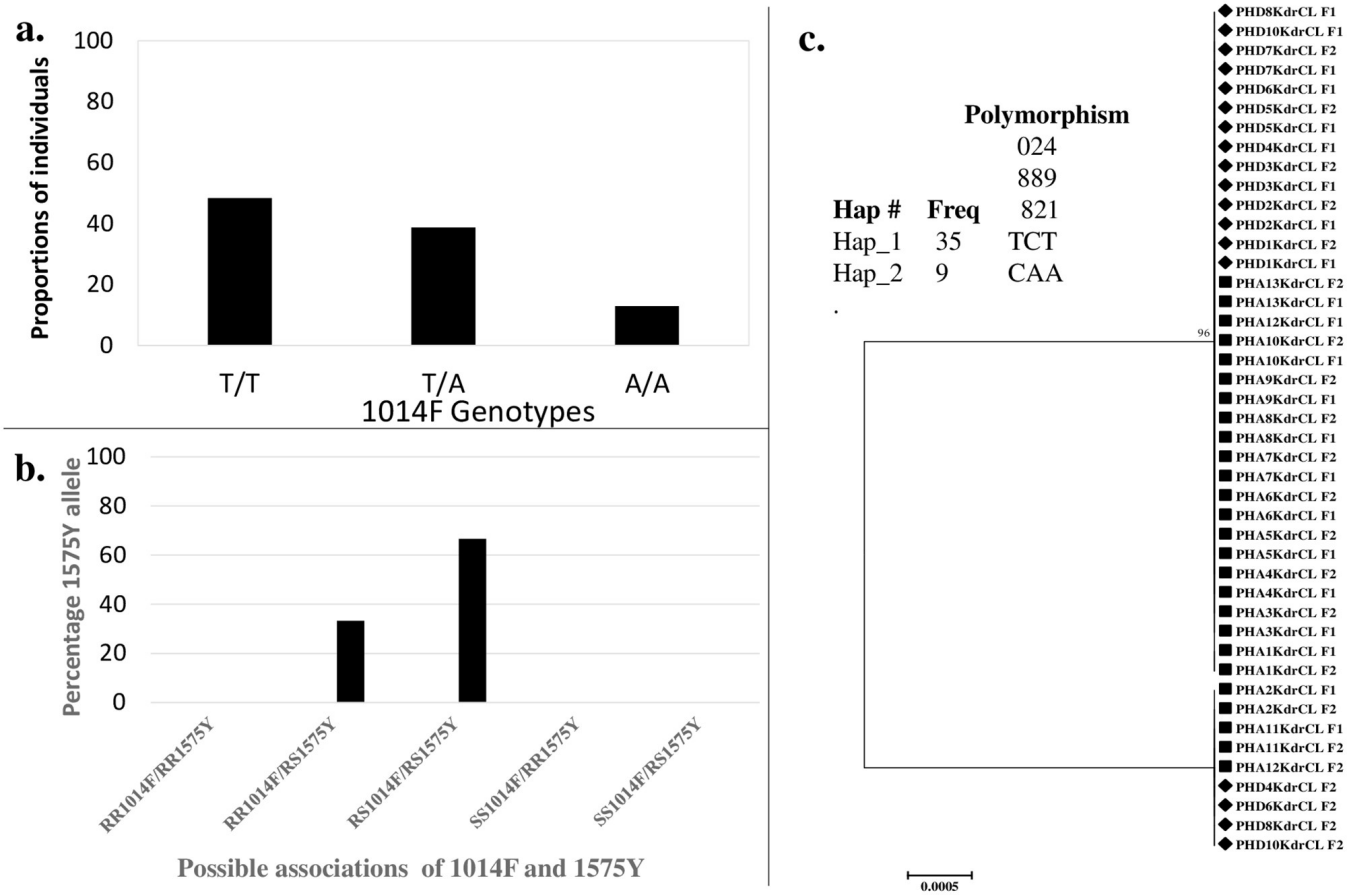
Determination of molecular basis of pyrethroid resistance in the *An*. *coluzzii* population. **a**. The genotype frequency distributions of the 1014F *kdr* mutation (T/T: homozygote resistant, T/A: heterozygote resistant and A/A: homozygote susceptible); **b**. Association between the 1575Y and the 1014F *kdr* mutations; **c**. maximum likelihood phylogenetic tree of fragment of VGSC from deltamethrin-alive (AL) and dead (DE) F_0_ female *An*. *coluzzii* from Port-Harcourt (PH). Inset: polymorphism analysis of a fragment of VGSC from deltamethrin-alive and dead F_0_ females showing only two haplotypes with variable positions.

Initial assessment of the presence of the *kdr* mutations in the VGSC of 32 field females revealed a high frequency of the 1014F mutation (0.84) and a very low frequency of the 1575Y mutation (0.1). No 1014S mutation was detected in all the females screened. For the 1014F *kdr* mutations, 48.4% of the individuals were homozygote resistant with the allele (RR) whereas 38.7% were heterozygote resistant (RS) (Fig 4a). Only 12.9% of the population harbour the wild type susceptible allele (SS). On the other hand, the 1575Y detected in low frequencies of ~10% was found only at heterozygote resistant (RS) state. The association between the 1014F and 1575Y mutations was such that 66% of 1575Y alleles were detected on heterozygote 1014F individuals (RS) while the 33% of the 1575Y alleles were detected on the 1014F homozygote resistant individuals (RR) (Fig 4b). This means 1575Y distribution might not be random but linked to RS 1014F. The 1575Y alleles were not detected on L1014 susceptible individuals.

To determine the genetic diversity of the VGSC a fragment encompassing the 1014 codon was amplified from 13 deltamethrin-resistant and 9 dead females. Analysis of a 548 bp fragment of VGSC revealed only two distinct haplotypes (Table 1, Fig 4c, S1 File), with low haplotype diversity of 0.33.

**Table 1:** Summary statistics of VGSC fragments in deltamethrin-alive and -dead *An*. *coluzzii* females.

Phenotype	n	S	h	Hd	π (k)	D(Tajima)	D* (Fu and Li)
Alive	13	3	2	0.323	0.00177 (0.969)	0.57140^ns^	0.96844^ns^
Dead	9	3	2	0.366	0.00220(1.09804)	0.7179^ns^	1.023060^ns^
All	22	3	2	0.333	0.00182 (0.9989)	0.95493^ns^	0.90523^ns^

n = number of sequences, S = polymorphic sites, h = haplotypes, Hd = haplotype diversity, π = nucleotide diversity (k = mean number of nucleotide differences); Tajima’s D and Fu and Li’s D* statistics, ns, not significant.

77% (10/13) of the deltamethrin-alive individuals were homozygote resistant while only 15% were homozygote susceptible in the same group. In contrast, only 55% of the deltamethrin-susceptible individuals were homozygote resistant and the remaining 45% were heterozygote resistant. No correlation was observed between deltamethrin resistance and the presence of the 1014F mutations, for example, RR+RS vs SS (Odds ratio = 0.61 (0.05–7.8, p = 0.70).

Haplotype 1 appeared to be the largest with ~80% of the sequences (35/44), whereas the haplotype 2 has the remaining 20%. Tajima’s D test of neutrality between the resistant and susceptible mosquitoes had a positive value but not significantly different. Moreover, both the deltamethrin-resistant and susceptible individuals appeared to have 3 polymorphic sites.

## Discussion

In this study resistance profile and the possible molecular mechanism behind it was assessed in the major malaria vector *An*. *coluzzii* from Niger-Delta of Nigeria, where malaria is stable/perennial [33]. Niger-Delta is the most polluted region in Nigeria due to crude oil related activities, such as gas flaring, illegal refineries, pipeline vandals and operational accidents leading to the release of pollutants into the environments in the forms of spillages [54]. These pollutants get washed into waters and the atmospheric spaces of the cities and villages within the region posing as additional selection pressure for insecticide resistance to disease vectors. The most recalcitrant oil related pollutants are the polycyclic aromatic hydrocarbons [55] which are also potent ligands for the aryl hydrocarbon receptor (AhR) involved in the transcriptional regulation of xenobiotic metabolizing enzymes in insects [56–58] and higher organisms [59]. The combinations of year-round breeding sites [33] and resistant vectors will be a good cocktail for malaria transmission. The *An*. *coluzzii* population from Port-Harcourt was found to be highly resistant to the pyrethroids and DDT with a very low mortalities for permethrin. In a previous study, resistance of > 25% mortality was reported in deltamethrin and DDT [60]. In the present work, mortalities of < 10% was seen with DDT and permethrin. In the south-eastern states of Nigeria which share some geographical features with the Niger-Delta, resistance to deltamethrin (57%) and DDT (13%) was recently reported [14]. While the above two studies [60] and [14] and our study reported full susceptibility to this bendiocarb observations made in *An*. *coluzzii* populations in the northern part of Nigeria suggest moderate resistance to this carbamate [15,16]. Moderate resistance to bendiocarb, may be attributed to the additional selection pressure from the use of carbamates in IRS and pesticides in agricultural fields [16,25]. In the neighbouring countries of Niger and Cameroun, despite not sharing the same geographical attributes with the Niger-Delta, *An*. *coluzzii* populations were also reported to be susceptible to malathion and bendiocarb [61,62].

The recovery of susceptibility following synergist bioassays suggests partial contribution of P450-mediated metabolic resistance in this population. The findings of *GSTe2*, a gene linked to DDT/pyrethroid resistance [43,44] overexpressed in the field population probably explained the alternative mechanism conferring pyrethroid resistance besides *kdr* mutation. The mortalities observed following pre-exposure to PBO was seven folds higher than the mortality recorded in the conventional bioassay. This shows a significant association between the PBO exposure and the increased in mortality even though the recovered susceptibility is ~50%. This is a trend that has been reported in recent studies [61,62] suggesting the potential of other detoxification mechanisms to the insecticides and/or other mechanisms including cuticular resistance on top of *kdr* resistance.

Transcriptional analysis of the candidate genes previously implicated in resistance to pyrethroids and DDT demonstrated overexpression of the genes pointing to potential roles of the metabolic resistance mechanisms. For example, recombinant protein of the *CYP6P3* (consistently established as overexpressed in field populations of *An*. *gambiae* s.l) was demonstrated to metabolize permethrin and deltamethrin in field resistant population [42,63,64]. Using transcriptional analysis this gene has previously been reported in pyrethroid resistant populations of *An*. *gambiae* in southwest Nigeria and Benin as overexpressed [24]. *CYP6Z2* linked to the pyrethroid resistance locus of *An*. *gambiae*, was reported to be involved in the metabolism of varying substrates including butein, resveratrol and entrodiol but not permethrin and cypermethrin [41]. However, *CYP6Z2* was found to metabolize the juvenile hormone analogue, pyriproxyfen in *An*. *gambiae* [65]. Though, pyriproxyfen was not tested on this population of *An*. *coluzzii* it is likely that the Niger-Delta population is resistant to pyriproxyfen which could reduce the efficacy of new LLINs incorporating this chemical. Overexpression of *GSTe2* has been linked with resistance to pyrethroid and DDT [43]. Not only in *An*. *gambiae* s.l. this gene is shown to be one of the major cross-resistance gene conferring resistance across diverse populations of *An*. *funestus* populations across Africa [44,66]. The rapid increase in resistance to pyrethroid in recent years has been attributed to the overexpression of multiple candidate genes in West Africa including the *CYP6Z3* [40] and the highly expressed *CYP6M3* reported in pyrethroid resistant populations of *An*. *gambiae* various parts of West Africa [39,67] as well as in *An*. *funestus* [68,69].

The 1014F *kdr* mutation was recorded at a high frequency of ~84% in the Niger-Delta population. Comparable frequency of ~ 82% have been reported in the northern part of Nigeria [15,16]. The highest frequency of 1014F *kdr* (88%) in Nigeria was reported in Niger state, north-central geopolitical zone [12]. Moreover, in the south eastern region, a frequency that ranges from 60% to 90% was detected [14]. In the neighbouring country of Niger, frequency ~82% was reported in the *An*. *coluzzii* [62]. However, a lower frequency of 60% was reported in Chad [62] and ~ 65% in Cameroon [61]. Looking at the frequencies of 1014F *kdr* mutations in parts of Nigeria and the neighbouring countries, it could be said that the mutation is approaching fixation; probably this explains the emergence of the 1575Y mutation in the populations to ameliorate the deleterious effects of the 1014F [49,70]. From the genotype data generated on this population, all the individuals detected were heterozygote with the 1575Y allele. It is interesting to mention here that all the alleles were detected on either heterozygote (RS) (66%) or homozygote resistant (RR) 1014F (33%). 1575Y alleles were not detected on any L1014 susceptible individuals, confirming the earlier projections that it always coexists with 1014F alleles thereby conferring additional advantages to the individuals. It was reported in the work of Jones and his associates, the1575Y *kdr* mutation, located in the exon 30 of the paratype VGSC coexists on the 1014F haplotypes, even though it was found to have an additive benefit, it is also alleged to compensate for the fitness cost lost due to 1014F in the field [49]. To the best of our knowledge this is the first study to document presence of this mutation in populations from Nigeria, though it has been documented in other parts of West Africa [49,71–73].

Analysis of the 548 bp fragment of the VGSC shows the VGSC is under selection pressure as a result of which it lead to the selection of only two distinct haplotypes within the population [74,75]. Low haplotype diversity (0.33) was observed between the deltamethrin-resistant and susceptible individuals and Tajma’s D* statistics also showed there was no significant difference between them.

## Conclusion

*Anopheles coluzzii* was the only species of *Anopheles* found in the various larval sampling sites in Port Harcourt. The population is highly resistant to pyrethroids and DDT, and fully susceptible to bendiocarb and malathion. Evidence of a role of metabolic resistance was shown possibly mediated by P450 monooxygenases and/or GSTs. High frequency of 1014F *kdr* mutation was seen and for the first time in a population of this major malaria vector from Nigeria presence of 1575Y *kdr* mutation established. Altogether, the findings of high pyrethroid resistance and the partial recovery with PBO suggest that pyrethroid-only LLINs may only have a reduced efficacy against these mosquitoes and that PBO-based nets should be considered. However, the failure to recover 100% mortality from PBO-synergist assay suggests that other insecticide classes should be considered in Port Harcourt including bendiocarb and malathion which could be used for indoor residual spraying.

## Supporting information

S1 Fig Agarose gel micrograph showing the 479 bp band pattern characteristic of *An*. *coluzzii* from *SINE200* PCR. Lane 1 = hyperladder IV (Bioline), 1013 bp. Lane 2–20 showing 479 bp with lane 4 and 16 showing no band.
(TIF)

S1 File Partial fragments of the voltage-gated sodium channel from deltamethrin-alive and dead *An*. *coluzzii* females. List of primers used for the qPCR and their respective sequences.
(DOCX)
