# Supplementary figures and images for: High pyrethroid/DDT resistance in major malaria vector Anopheles coluzzii from Niger-Delta of Nigeria is probably driven by metabolic resistance mechanisms
Source: PLoS One. 2021 Mar 11;16(3):e0247944. doi: 10.1371/journal.pone.0247944 (PMC7951933; doi:10.1371/journal.pone.0247944)

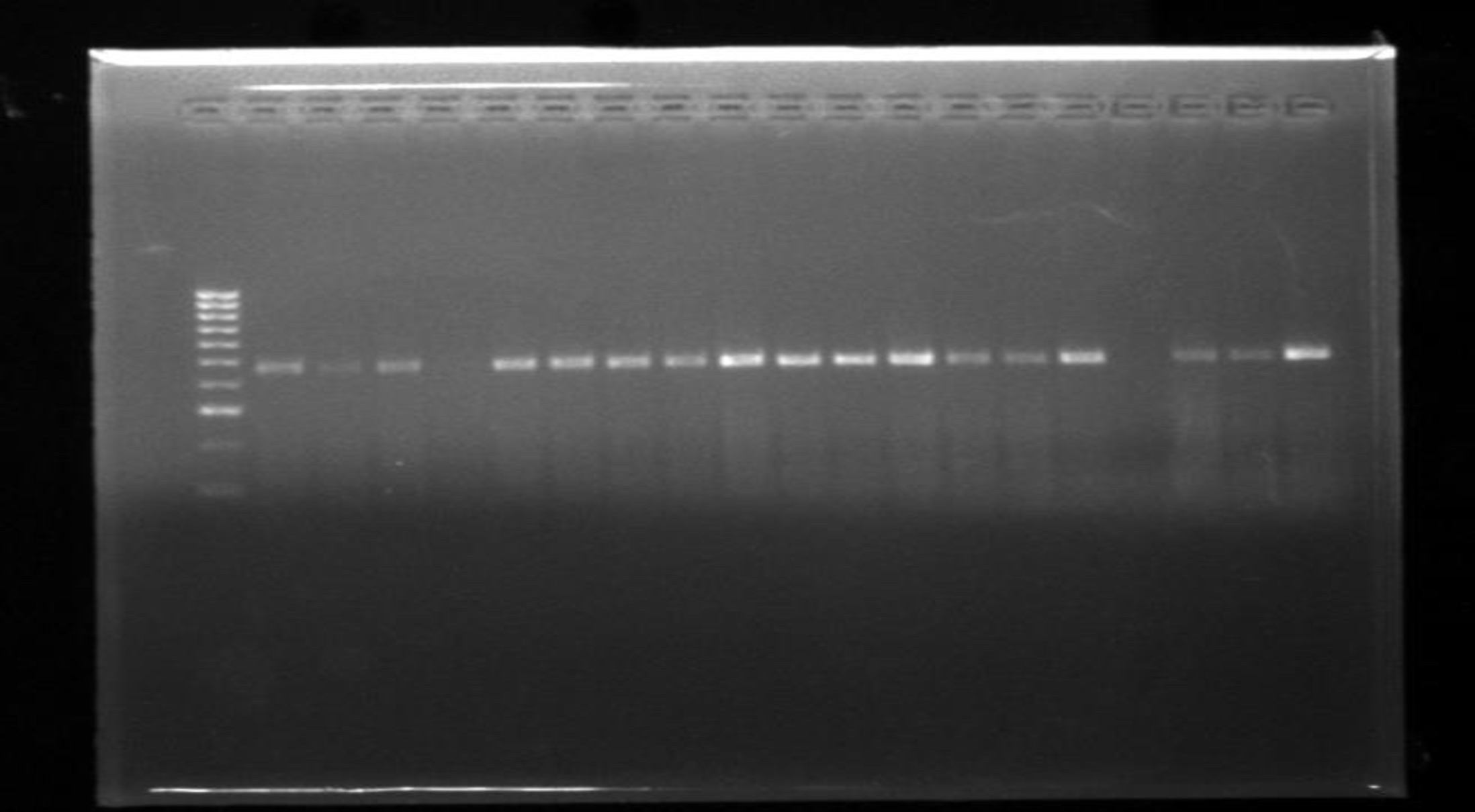

Supplement: S1 Fig — Lane 1 = hyperladder IV (Bioline), 1013 bp. Lane 2–20 showing 479 bp with lane 4 and 16 showing no band. (TIF) [file pone.0247944.s001.tif]
