# Supplementary material for: High pyrethroid/DDT resistance in major malaria vector Anopheles coluzzii from Niger-Delta of Nigeria is probably driven by metabolic resistance mechanisms
Source: PLoS One. 2021 Mar 11;16(3):e0247944. doi: 10.1371/journal.pone.0247944 (PMC7951933; doi:10.1371/journal.pone.0247944)
Supplement: S1 File — List of primers used for the qPCR and their respective sequences. (DOCX) [file pone.0247944.s002.docx]

| **Candidate genes** | **Forward Primer** | | **Reverse Primer** | **Accession number** | **Reference** |
| --- | --- | --- | --- | --- | --- |
| *GSTe2* | ACCATTAATCTGCTAACGGGTG | | AATTTACACGGGCCTGCTTG | [ACOM042596](https://www.vectorbase.org/Anopheles_coluzzii/Gene/Compara_Ortholog?g=ACOM042596) | 1(Toé *et al.*, 2015) |
| *CYP6Z3* | CTGGTCCACGCAATTGCATTGGTCT | | ACGCGCATGGGGAAACCATCTT | [ACOM030346](https://www.vectorbase.org/Anopheles_coluzzii/Gene/Summary/?g=ACOM030346) | (Toé *et al.*, 2015) |
| *CYP6P3* | AGCGATGCTTTGTTTTGCTT | | AGCGATGCTTTGTTTTGCTT | [ACOM037567](https://www.vectorbase.org/Anopheles_coluzzii/Gene/Summary/?g=ACOM037567) | (Kwiatkowska *et al.*, 2013) |
| *CYP6Z2* | AGTTCAAGTTCCAGGCCACA | TTTAGTTCATCACAATCAGTTGC | | [AGAP008218](https://www.vectorbase.org/Anopheles_gambiae/Gene/Summary/?g=AGAP008218) | (Kwiatkowska *et al.*, 2013) |
| *CYP6M3* | TGGACCAGATACTGAAGGAGAGT | | ACAGAGGTTCCTGCTTCGAG | [ACOM043129](https://www.vectorbase.org/Anopheles_arabiensis/Gene/Compara_Ortholog?g=AARA015642) | (Kwiatkowska *et al.*, 2013) |
| *RSP7* | GTGTTCGGTTCCAAGGTGAT | | TCCGAGTTCATTTCCAGCTC | AGAP010592 | (Kwiatkowska *et al.*, 2013) |
| *GADPH* | CTGCAAAAAGTCGATACCGC | | CCTCGTACACGTACATCGTGA | ACOM032621 | (Dimopoulos *et al*., 1996) |

**S1 Table**: **List of primer sequences used in the qPCR**

**Partial fragment sequence of the voltage-gated sodium channel from deltamethrin-alive and dead *An. coluzzii* females.**

>PHA1-KdrCL_F 548 bp

AAATCAGCCAAAATTTTCTTGCAATTGGTTTGTAACTATATTATTTGCAA

TGTACATGCATTATGCTCTTTACAATGCCAACGCAATTCCTGTTAAAGAA

AATGCATTAACGATAAGCTTTTAGAAAAAGGTTTAGAGAAACAGCTTACA

ATGTCTTGATGATCAATTCAAGTAGTCTACTAGCTTATTCTCAATTATTA

TATATTCACTAACGCGAATTAAATGCTTTGTGACAGATTTCATCAGAAAA

TCAGTGTTTTGCTAGCCTAATTGCTTTTTTCCTTTTCTTTAATATACTTT

TTCCAGATAATGTGGATAGATTCCCCGACCATGATCTGCCAAGATGGAAT

TTTACAGATTTCATGCATTCCTTCATGATTGTGTTCCGTGTGCTATGCGG

AGAATGGATTGAATCAATGTGGGATTGTATGCTTGTCGGTGATGTATCCT

GCATACCATTTTTCTTGGCCACTGTAGTGATAGGAAATTTTGTCGTAAGT

AATGCAAATTAACATGGACCAAGATCGTTTTTACATGACATTGTTTTG

>PHA2-KdrCL_F 548 bp

AAATCAGCCAAAATTTTCTTGCAATTGGTTTGTAACTATATTATTTGCAA

TGTACATGCATTATGCTCTTTACAATGCCAACGCAATCCCTGTTAAAGAA

AATGCATTAACGATAAGCTTTTAGAAAAAGGTTTAGAGAAACAGCTTACA

ATGTCTTGATGATCAATTCAAGTAGTCTACTAGCTTATTCTCAATTATTA

TATATTCACTAACGCGAATTAAATGCTTTGTGACAGATTTCATCAGAAAA

TCAGTGTTTTGCTAGCCTAATTGCTTTTTTCATTTTCTTTAATATACTTT

TTCCAGATAATGTGGATAGATTCCCCGACCATGATCTGCCAAGATGGAAT

TTTACAGATTTCATGCATTCCTTCATGATTGTGTTCCGTGTGCTATGCGG

AGAATGGATTGAATCAATGTGGGATTGTATGCTTGTCGGTGATGTATCCT

GCATACCATTTTTCTTGGCCACTGTAGTGATAGGAAATTTAGTCGTAAGT

AATGCAAATTAACATGGACCAAGATCGTTTTTACATGACATTGTTTTG

>PHA3-KdrCL_F 548 bp

AAATCAGCCAAAATTTTCTTGCAATTGGTTTGTAACTATATTATTTGCAA

TGTACATGCATTATGCTCTTTACAATGCCAACGCAATTCCTGTTAAAGAA

AATGCATTAACGATAAGCTTTTAGAAAAAGGTTTAGAGAAACAGCTTACA

ATGTCTTGATGATCAATTCAAGTAGTCTACTAGCTTATTCTCAATTATTA

TATATTCACTAACGCGAATTAAATGCTTTGTGACAGATTTCATCAGAAAA

TCAGTGTTTTGCTAGCCTAATTGCTTTTTTCCTTTTCTTTAATATACTTT

TTCCAGATAATGTGGATAGATTCCCCGACCATGATCTGCCAAGATGGAAT

TTTACAGATTTCATGCATTCCTTCATGATTGTGTTCCGTGTGCTATGCGG

AGAATGGATTGAATCAATGTGGGATTGTATGCTTGTCGGTGATGTATCCT

GCATACCATTTTTCTTGGCCACTGTAGTGATAGGAAATTTTGTCGTAAGT

AATGCAAATTAACATGGACCAAGATCGTTTTTACATGACATTGTTTTG

>PHA4-KdrCL_F 548 bp

AAATCAGCCAAAATTTTCTTGCAATTGGTTTGTAACTATATTATTTGCAA

TGTACATGCATTATGCTCTTTACAATGCCAACGCAATTCCTGTTAAAGAA

AATGCATTAACGATAAGCTTTTAGAAAAAGGTTTAGAGAAACAGCTTACA

ATGTCTTGATGATCAATTCAAGTAGTCTACTAGCTTATTCTCAATTATTA

TATATTCACTAACGCGAATTAAATGCTTTGTGACAGATTTCATCAGAAAA

TCAGTGTTTTGCTAGCCTAATTGCTTTTTTCCTTTTCTTTAATATACTTT

TTCCAGATAATGTGGATAGATTCCCCGACCATGATCTGCCAAGATGGAAT

TTTACAGATTTCATGCATTCCTTCATGATTGTGTTCCGTGTGCTATGCGG

AGAATGGATTGAATCAATGTGGGATTGTATGCTTGTCGGTGATGTATCCT

GCATACCATTTTTCTTGGCCACTGTAGTGATAGGAAATTTTGTCGTAAGT

AATGCAAATTAACATGGACCAAGATCGTTTTTACATGACATTGTTTTG

>PHA5-KdrCL_F 548 bp

AAATCAGCCAAAATTTTCTTGCAATTGGTTTGTAACTATATTATTTGCAA

TGTACATGCATTATGCTCTTTACAATGCCAACGCAATTCCTGTTAAAGAA

AATGCATTAACGATAAGCTTTTAGAAAAAGGTTTAGAGAAACAGCTTACA

ATGTCTTGATGATCAATTCAAGTAGTCTACTAGCTTATTCTCAATTATTA

TATATTCACTAACGCGAATTAAATGCTTTGTGACAGATTTCATCAGAAAA

TCAGTGTTTTGCTAGCCTAATTGCTTTTTTCCTTTTCTTTAATATACTTT

TTCCAGATAATGTGGATAGATTCCCCGACCATGATCTGCCAAGATGGAAT

TTTACAGATTTCATGCATTCCTTCATGATTGTGTTCCGTGTGCTATGCGG

AGAATGGATTGAATCAATGTGGGATTGTATGCTTGTCGGTGATGTATCCT

GCATACCATTTTTCTTGGCCACTGTAGTGATAGGAAATTTTGTCGTAAGT

AATGCAAATTAACATGGACCAAGATCGTTTTTACATGACATTGTTTTG

>PHA6-KdrCL_F 548 bp

AAATCAGCCAAAATTTTCTTGCAATTGGTTTGTAACTATATTATTTGCAA

TGTACATGCATTATGCTCTTTACAATGCCAACGCAATTCCTGTTAAAGAA

AATGCATTAACGATAAGCTTTTAGAAAAAGGTTTAGAGAAACAGCTTACA

ATGTCTTGATGATCAATTCAAGTAGTCTACTAGCTTATTCTCAATTATTA

TATATTCACTAACGCGAATTAAATGCTTTGTGACAGATTTCATCAGAAAA

TCAGTGTTTTGCTAGCCTAATTGCTTTTTTCCTTTTCTTTAATATACTTT

TTCCAGATAATGTGGATAGATTCCCCGACCATGATCTGCCAAGATGGAAT

TTTACAGATTTCATGCATTCCTTCATGATTGTGTTCCGTGTGCTATGCGG

AGAATGGATTGAATCAATGTGGGATTGTATGCTTGTCGGTGATGTATCCT

GCATACCATTTTTCTTGGCCACTGTAGTGATAGGAAATTTTGTCGTAAGT

AATGCAAATTAACATGGACCAAGATCGTTTTTACATGACATTGTTTTG

>PHA7-KdrCL_F 548 bp

AAATCAGCCAAAATTTTCTTGCAATTGGTTTGTAACTATATTATTTGCAA

TGTACATGCATTATGCTCTTTACAATGCCAACGCAATTCCTGTTAAAGAA

AATGCATTAACGATAAGCTTTTAGAAAAAGGTTTAGAGAAACAGCTTACA

ATGTCTTGATGATCAATTCAAGTAGTCTACTAGCTTATTCTCAATTATTA

TATATTCACTAACGCGAATTAAATGCTTTGTGACAGATTTCATCAGAAAA

TCAGTGTTTTGCTAGCCTAATTGCTTTTTTCCTTTTCTTTAATATACTTT

TTCCAGATAATGTGGATAGATTCCCCGACCATGATCTGCCAAGATGGAAT

TTTACAGATTTCATGCATTCCTTCATGATTGTGTTCCGTGTGCTATGCGG

AGAATGGATTGAATCAATGTGGGATTGTATGCTTGTCGGTGATGTATCCT

GCATACCATTTTTCTTGGCCACTGTAGTGATAGGAAATTTTGTCGTAAGT

AATGCAAATTAACATGGACCAAGATCGTTTTTACATGACATTGTTTTG

>PHA8-KdrCL_F 548 bp

AAATCAGCCAAAATTTTCTTGCAATTGGTTTGTAACTATATTATTTGCAA

TGTACATGCATTATGCTCTTTACAATGCCAACGCAATTCCTGTTAAAGAA

AATGCATTAACGATAAGCTTTTAGAAAAAGGTTTAGAGAAACAGCTTACA

ATGTCTTGATGATCAATTCAAGTAGTCTACTAGCTTATTCTCAATTATTA

TATATTCACTAACGCGAATTAAATGCTTTGTGACAGATTTCATCAGAAAA

TCAGTGTTTTGCTAGCCTAATTGCTTTTTTCCTTTTCTTTAATATACTTT

TTCCAGATAATGTGGATAGATTCCCCGACCATGATCTGCCAAGATGGAAT

TTTACAGATTTCATGCATTCCTTCATGATTGTGTTCCGTGTGCTATGCGG

AGAATGGATTGAATCAATGTGGGATTGTATGCTTGTCGGTGATGTATCCT

GCATACCATTTTTCTTGGCCACTGTAGTGATAGGAAATTTTGTCGTAAGT

AATGCAAATTAACATGGACCAAGATCGTTTTTACATGACATTGTTTTG

>PHA9-KdrCL_F 548 bp

AAATCAGCCAAAATTTTCTTGCAATTGGTTTGTAACTATATTATTTGCAA

TGTACATGCATTATGCTCTTTACAATGCCAACGCAATTCCTGTTAAAGAA

AATGCATTAACGATAAGCTTTTAGAAAAAGGTTTAGAGAAACAGCTTACA

ATGTCTTGATGATCAATTCAAGTAGTCTACTAGCTTATTCTCAATTATTA

TATATTCACTAACGCGAATTAAATGCTTTGTGACAGATTTCATCAGAAAA

TCAGTGTTTTGCTAGCCTAATTGCTTTTTTCCTTTTCTTTAATATACTTT

TTCCAGATAATGTGGATAGATTCCCCGACCATGATCTGCCAAGATGGAAT

TTTACAGATTTCATGCATTCCTTCATGATTGTGTTCCGTGTGCTATGCGG

AGAATGGATTGAATCAATGTGGGATTGTATGCTTGTCGGTGATGTATCCT

GCATACCATTTTTCTTGGCCACTGTAGTGATAGGAAATTTTGTCGTAAGT

AATGCAAATTAACATGGACCAAGATCGTTTTTACATGACATTGTTTTG

>PHA10-KdrCL_F 548 bp

AAATCAGCCAAAATTTTCTTGCAATTGGTTTGTAACTATATTATTTGCAA

TGTACATGCATTATGCTCTTTACAATGCCAACGCAATTCCTGTTAAAGAA

AATGCATTAACGATAAGCTTTTAGAAAAAGGTTTAGAGAAACAGCTTACA

ATGTCTTGATGATCAATTCAAGTAGTCTACTAGCTTATTCTCAATTATTA

TATATTCACTAACGCGAATTAAATGCTTTGTGACAGATTTCATCAGAAAA

TCAGTGTTTTGCTAGCCTAATTGCTTTTTTCCTTTTCTTTAATATACTTT

TTCCAGATAATGTGGATAGATTCCCCGACCATGATCTGCCAAGATGGAAT

TTTACAGATTTCATGCATTCCTTCATGATTGTGTTCCGTGTGCTATGCGG

AGAATGGATTGAATCAATGTGGGATTGTATGCTTGTCGGTGATGTATCCT

GCATACCATTTTTCTTGGCCACTGTAGTGATAGGAAATTTTGTCGTAAGT

AATGCAAATTAACATGGACCAAGATCGTTTTTACATGACATTGTTTTG

>PHA11-KdrCL_F 548 bp

AAATCAGCCAAAATTTTCTTGCAATTGGTTTGTAACTATATTATTTGCAA

TGTACATGCATTATGCTCTTTACAATGCCAACGCAATCCCTGTTAAAGAA

AATGCATTAACGATAAGCTTTTAGAAAAAGGTTTAGAGAAACAGCTTACA

ATGTCTTGATGATCAATTCAAGTAGTCTACTAGCTTATTCTCAATTATTA

TATATTCACTAACGCGAATTAAATGCTTTGTGACAGATTTCATCAGAAAA

TCAGTGTTTTGCTAGCCTAATTGCTTTTTTCATTTTCTTTAATATACTTT

TTCCAGATAATGTGGATAGATTCCCCGACCATGATCTGCCAAGATGGAAT

TTTACAGATTTCATGCATTCCTTCATGATTGTGTTCCGTGTGCTATGCGG

AGAATGGATTGAATCAATGTGGGATTGTATGCTTGTCGGTGATGTATCCT

GCATACCATTTTTCTTGGCCACTGTAGTGATAGGAAATTTAGTCGTAAGT

AATGCAAATTAACATGGACCAAGATCGTTTTTACATGACATTGTTTTG

>PHA12-KdrCL_F 548 bp

AAATCAGCCAAAATTTTCTTGCAATTGGTTTGTAACTATATTATTTGCAA

TGTACATGCATTATGCTCTTTACAATGCCAACGCAATYCCTGTTAAAGAA

AATGCATTAACGATAAGCTTTTAGAAAAAGGTTTAGAGAAACAGCTTACA

ATGTCTTGATGATCAATTCAAGTAGTCTACTAGCTTATTCTCAATTATTA

TATATTCACTAACGCGAATTAAATGCTTTGTGACAGATTTCATCAGAAAA

TCAGTGTTTTGCTAGCCTAATTGCTTTTTTCMTTTTCTTTAATATACTTT

TTCCAGATAATGTGGATAGATTCCCCGACCATGATCTGCCAAGATGGAAT

TTTACAGATTTCATGCATTCCTTCATGATTGTGTTCCGTGTGCTATGCGG

AGAATGGATTGAATCAATGTGGGATTGTATGCTTGTCGGTGATGTATCCT

GCATACCATTTTTCTTGGCCACTGTAGTGATAGGAAATTTWGTCGTAAGT

AATGCAAATTAACATGGACCAAGATCGTTTTTACATGACATTGTTTTG

>PHA13-KdrCL_F 548 bp

AAATCAGCCAAAATTTTCTTGCAATTGGTTTGTAACTATATTATTTGCAA

TGTACATGCATTATGCTCTTTACAATGCCAACGCAATTCCTGTTAAAGAA

AATGCATTAACGATAAGCTTTTAGAAAAAGGTTTAGAGAAACAGCTTACA

ATGTCTTGATGATCAATTCAAGTAGTCTACTAGCTTATTCTCAATTATTA

TATATTCACTAACGCGAATTAAATGCTTTGTGACAGATTTCATCAGAAAA

TCAGTGTTTTGCTAGCCTAATTGCTTTTTTCCTTTTCTTTAATATACTTT

TTCCAGATAATGTGGATAGATTCCCCGACCATGATCTGCCAAGATGGAAT

TTTACAGATTTCATGCATTCCTTCATGATTGTGTTCCGTGTGCTATGCGG

AGAATGGATTGAATCAATGTGGGATTGTATGCTTGTCGGTGATGTATCCT

GCATACCATTTTTCTTGGCCACTGTAGTGATAGGAAATTTTGTCGTAAGT

AATGCAAATTAACATGGACCAAGATCGTTTTTACATGACATTGTTTTG

>PHD1-KdrCL_F 548 bp

AAATCAGCCAAAATTTTCTTGCAATTGGTTTGTAACTATATTATTTGCAA

TGTACATGCATTATGCTCTTTACAATGCCAACGCAATTCCTGTTAAAGAA

AATGCATTAACGATAAGCTTTTAGAAAAAGGTTTAGAGAAACAGCTTACA

ATGTCTTGATGATCAATTCAAGTAGTCTACTAGCTTATTCTCAATTATTA

TATATTCACTAACGCGAATTAAATGCTTTGTGACAGATTTCATCAGAAAA

TCAGTGTTTTGCTAGCCTAATTGCTTTTTTCCTTTTCTTTAATATACTTT

TTCCAGATAATGTGGATAGATTCCCCGACCATGATCTGCCAAGATGGAAT

TTTACAGATTTCATGCATTCCTTCATGATTGTGTTCCGTGTGCTATGCGG

AGAATGGATTGAATCAATGTGGGATTGTATGCTTGTCGGTGATGTATCCT

GCATACCATTTTTCTTGGCCACTGTAGTGATAGGAAATTTTGTCGTAAGT

AATGCAAATTAACATGGACCAAGATCGTTTTTACATGACATTGTTTTG

>PHD2-KdrCL_F 548 bp

AAATCAGCCAAAATTTTCTTGCAATTGGTTTGTAACTATATTATTTGCAA

TGTACATGCATTATGCTCTTTACAATGCCAACGCAATTCCTGTTAAAGAA

AATGCATTAACGATAAGCTTTTAGAAAAAGGTTTAGAGAAACAGCTTACA

ATGTCTTGATGATCAATTCAAGTAGTCTACTAGCTTATTCTCAATTATTA

TATATTCACTAACGCGAATTAAATGCTTTGTGACAGATTTCATCAGAAAA

TCAGTGTTTTGCTAGCCTAATTGCTTTTTTCCTTTTCTTTAATATACTTT

TTCCAGATAATGTGGATAGATTCCCCGACCATGATCTGCCAAGATGGAAT

TTTACAGATTTCATGCATTCCTTCATGATTGTGTTCCGTGTGCTATGCGG

AGAATGGATTGAATCAATGTGGGATTGTATGCTTGTCGGTGATGTATCCT

GCATACCATTTTTCTTGGCCACTGTAGTGATAGGAAATTTTGTCGTAAGT

AATGCAAATTAACATGGACCAAGATCGTTTTTACATGACATTGTTTTG

>PHD3-KdrCL_F 548 bp

AAATCAGCCAAAATTTTCTTGCAATTGGTTTGTAACTATATTATTTGCAA

TGTACATGCATTATGCTCTTTACAATGCCAACGCAATTCCTGTTAAAGAA

AATGCATTAACGATAAGCTTTTAGAAAAAGGTTTAGAGAAACAGCTTACA

ATGTCTTGATGATCAATTCAAGTAGTCTACTAGCTTATTCTCAATTATTA

TATATTCACTAACGCGAATTAAATGCTTTGTGACAGATTTCATCAGAAAA

TCAGTGTTTTGCTAGCCTAATTGCTTTTTTCCTTTTCTTTAATATACTTT

TTCCAGATAATGTGGATAGATTCCCCGACCATGATCTGCCAAGATGGAAT

TTTACAGATTTCATGCATTCCTTCATGATTGTGTTCCGTGTGCTATGCGG

AGAATGGATTGAATCAATGTGGGATTGTATGCTTGTCGGTGATGTATCCT

GCATACCATTTTTCTTGGCCACTGTAGTGATAGGAAATTTTGTCGTAAGT

AATGCAAATTAACATGGACCAAGATCGTTTTTACATGACATTGTTTTG

>PHD4-KdrCL_F 548 bp

AAATCAGCCAAAATTTTCTTGCAATTGGTTTGTAACTATATTATTTGCAA

TGTACATGCATTATGCTCTTTACAATGCCAACGCAATYCCTGTTAAAGAA

AATGCATTAACGATAAGCTTTTAGAAAAAGGTTTAGAGAAACAGCTTACA

ATGTCTTGATGATCAATTCAAGTAGTCTACTAGCTTATTCTCAATTATTA

TATATTCACTAACGCGAATTAAATGCTTTGTGACAGATTTCATCAGAAAA

TCAGTGTTTTGCTAGCCTAATTGCTTTTTTCMTTTTCTTTAATATACTTT

TTCCAGATAATGTGGATAGATTCCCCGACCATGATCTGCCAAGATGGAAT

TTTACAGATTTCATGCATTCCTTCATGATTGTGTTCCGTGTGCTATGCGG

AGAATGGATTGAATCAATGTGGGATTGTATGCTTGTCGGTGATGTATCCT

GCATACCATTTTTCTTGGCCACTGTAGTGATAGGAAATTTWGTCGTAAGT

AATGCAAATTAACATGGACCAAGATCGTTTTTACATGACATTGTTTTG

>PHD5-KdrCL_F 548 bp

AAATCAGCCAAAATTTTCTTGCAATTGGTTTGTAACTATATTATTTGCAA

TGTACATGCATTATGCTCTTTACAATGCCAACGCAATTCCTGTTAAAGAA

AATGCATTAACGATAAGCTTTTAGAAAAAGGTTTAGAGAAACAGCTTACA

ATGTCTTGATGATCAATTCAAGTAGTCTACTAGCTTATTCTCAATTATTA

TATATTCACTAACGCGAATTAAATGCTTTGTGACAGATTTCATCAGAAAA

TCAGTGTTTTGCTAGCCTAATTGCTTTTTTCCTTTTCTTTAATATACTTT

TTCCAGATAATGTGGATAGATTCCCCGACCATGATCTGCCAAGATGGAAT

TTTACAGATTTCATGCATTCCTTCATGATTGTGTTCCGTGTGCTATGCGG

AGAATGGATTGAATCAATGTGGGATTGTATGCTTGTCGGTGATGTATCCT

GCATACCATTTTTCTTGGCCACTGTAGTGATAGGAAATTTTGTCGTAAGT

AATGCAAATTAACATGGACCAAGATCGTTTTTACATGACATTGTTTTG

>PHD6-KdrCL_F 548 bp

AAATCAGCCAAAATTTTCTTGCAATTGGTTTGTAACTATATTATTTGCAA

TGTACATGCATTATGCTCTTTACAATGCCAACGCAATYCCTGTTAAAGAA

AATGCATTAACGATAAGCTTTTAGAAAAAGGTTTAGAGAAACAGCTTACA

ATGTCTTGATGATCAATTCAAGTAGTCTACTAGCTTATTCTCAATTATTA

TATATTCACTAACGCGAATTAAATGCTTTGTGACAGATTTCATCAGAAAA

TCAGTGTTTTGCTAGCCTAATTGCTTTTTTCMTTTTCTTTAATATACTTT

TTCCAGATAATGTGGATAGATTCCCCGACCATGATCTGCCAAGATGGAAT

TTTACAGATTTCATGCATTCCTTCATGATTGTGTTCCGTGTGCTATGCGG

AGAATGGATTGAATCAATGTGGGATTGTATGCTTGTCGGTGATGTATCCT

GCATACCATTTTTCTTGGCCACTGTAGTGATAGGAAATTTWGTCGTAAGT

AATGCAAATTAACATGGACCAAGATCGTTTTTACATGACATTGTTTTG

>PHD7-KdrCL_F 548 bp

AAATCAGCCAAAATTTTCTTGCAATTGGTTTGTAACTATATTATTTGCAA

TGTACATGCATTATGCTCTTTACAATGCCAACGCAATTCCTGTTAAAGAA

AATGCATTAACGATAAGCTTTTAGAAAAAGGTTTAGAGAAACAGCTTACA

ATGTCTTGATGATCAATTCAAGTAGTCTACTAGCTTATTCTCAATTATTA

TATATTCACTAACGCGAATTAAATGCTTTGTGACAGATTTCATCAGAAAA

TCAGTGTTTTGCTAGCCTAATTGCTTTTTTCCTTTTCTTTAATATACTTT

TTCCAGATAATGTGGATAGATTCCCCGACCATGATCTGCCAAGATGGAAT

TTTACAGATTTCATGCATTCCTTCATGATTGTGTTCCGTGTGCTATGCGG

AGAATGGATTGAATCAATGTGGGATTGTATGCTTGTCGGTGATGTATCCT

GCATACCATTTTTCTTGGCCACTGTAGTGATAGGAAATTTTGTCGTAAGT

AATGCAAATTAACATGGACCAAGATCGTTTTTACATGACATTGTTTTG

>PHD8-KdrCL_F 548 bp

AAATCAGCCAAAATTTTCTTGCAATTGGTTTGTAACTATATTATTTGCAA

TGTACATGCATTATGCTCTTTACAATGCCAACGCAATYCCTGTTAAAGAA

AATGCATTAACGATAAGCTTTTAGAAAAAGGTTTAGAGAAACAGCTTACA

ATGTCTTGATGATCAATTCAAGTAGTCTACTAGCTTATTCTCAATTATTA

TATATTCACTAACGCGAATTAAATGCTTTGTGACAGATTTCATCAGAAAA

TCAGTGTTTTGCTAGCCTAATTGCTTTTTTCMTTTTCTTTAATATACTTT

TTCCAGATAATGTGGATAGATTCCCCGACCATGATCTGCCAAGATGGAAT

TTTACAGATTTCATGCATTCCTTCATGATTGTGTTCCGTGTGCTATGCGG

AGAATGGATTGAATCAATGTGGGATTGTATGCTTGTCGGTGATGTATCCT

GCATACCATTTTTCTTGGCCACTGTAGTGATAGGAAATTTWGTCGTAAGT

AATGCAAATTAACATGGACCAAGATCGTTTTTACATGACATTGTTTTG

>PHD10-KdrCL_F 548 bp

AAATCAGCCAAAATTTTCTTGCAATTGGTTTGTAACTATATTATTTGCAA

TGTACATGCATTATGCTCTTTACAATGCCAACGCAATYCCTGTTAAAGAA

AATGCATTAACGATAAGCTTTTAGAAAAAGGTTTAGAGAAACAGCTTACA

ATGTCTTGATGATCAATTCAAGTAGTCTACTAGCTTATTCTCAATTATTA

TATATTCACTAACGCGAATTAAATGCTTTGTGACAGATTTCATCAGAAAA

TCAGTGTTTTGCTAGCCTAATTGCTTTTTTCMTTTTCTTTAATATACTTT

TTCCAGATAATGTGGATAGATTCCCCGACCATGATCTGCCAAGATGGAAT

TTTACAGATTTCATGCATTCCTTCATGATTGTGTTCCGTGTGCTATGCGG

AGAATGGATTGAATCAATGTGGGATTGTATGCTTGTCGGTGATGTATCCT

GCATACCATTTTTCTTGGCCACTGTAGTGATAGGAAATTTWGTCGTAAGT

AATGCAAATTAACATGGACCAAGATCGTTTTTACATGACATTGTTTTG
